# Dynamical and Mechanistic Reconstructive Approaches of T Lymphocyte Dynamics: Using Visual Modeling Languages to Bridge the Gap between Immunologists, Theoreticians, and Programmers

**DOI:** 10.3389/fimmu.2013.00300

**Published:** 2013-10-01

**Authors:** Véronique Thomas-Vaslin, Adrien Six, Jean-Gabriel Ganascia, Hugues Bersini

**Affiliations:** ^1^UPMC Univ Paris 06, UMR7211, Immunology, Immunopathology, Immunotherapy (I3), Integrative Immunology, Paris, France; ^2^CNRS, UMR 7211, Immunology-Immunopathology-Immunotherapy (I3) Integrative Immunology, Paris, France; ^3^UPMC Univ Paris 06, UMR 7606, ACASA-LIP6, Paris, France; ^4^CNRS, UMR 7606, ACASA-LIP6, CNRS, Paris, France; ^5^Université Libre de Bruxelles, IRIDIA-Code, Bruxelles, Belgium

**Keywords:** state-transition diagram, computer modeling, cell dynamics, agent-based model, complex system

## Abstract

Dynamic modeling of lymphocyte behavior has primarily been based on populations based differential equations or on cellular agents moving in space and interacting each other. The final steps of this modeling effort are expressed in a code written in a programing language. On account of the complete lack of standardization of the different steps to proceed, we have to deplore poor communication and sharing between experimentalists, theoreticians and programmers. The adoption of diagrammatic visual computer language should however greatly help the immunologists to better communicate, to more easily identify the models similarities and facilitate the reuse and extension of existing software models. Since immunologists often conceptualize the dynamical evolution of immune systems in terms of “state-transitions” of biological objects, we promote the use of unified modeling language (UML) state-transition diagram. To demonstrate the feasibility of this approach, we present a UML refactoring of two published models on thymocyte differentiation. Originally built with different modeling strategies, a mathematical ordinary differential equation-based model and a cellular automata model, the two models are now in the same visual formalism and can be compared.

The perspective is to encourage immunologists involved into mathematical modeling or software productions, to adopt a visual graphical language, here mainly the unified modeling language (UML) “state-transition” diagram to ease the communication, the reuse and the extension of their models.

## Complexity of the Immune System

The immune system is a complex biological adaptive, highly diversified, robust and resilient system, characterized by complexity at different levels. Lymphocytes are the central actors of the immune system, in the middle of a multi-scale biological organization, “from molecule to organism”. Multi-scale modeling remains a challenge, as for other biological systems (1). Despite recent systems biology initiatives to understand and model the immune system (2), we are still far from having the appropriate tools to understand its dynamics and to easily communicate among various researchers who observe this system at different levels of granularity and attempt through software modeling to answer different questions. Several complementary experimental methods and models have been used to explore lymphocyte dynamics and turnover (3, 4) and to model it in health, aging and diseases (5).

## Drawbacks of Current Dynamics Lymphocyte Modeling and Evolution

System dynamics models deal with time, formalized with two distinct concepts as “discrete time,” by a succession of time points and intervals, or as “continuous time” (6). Models of lymphocyte population dynamics and turnover (3) have primarily been based on mechanistic reconstruction with continuous time models. The fluxes of cell populations are then described by differential equations. These mathematical models describe for example the thymocyte differentiation and selection (7), until the thymic export (8–10), the homeostasis of CD4/CD8 T cells (11, 12), the CD8 immune response (13, 14), or the Bromodeoxyuridine or deuterium labeling (15) to account for turnover. Up to now, simulations and validation of some of these models reveal interesting T cell dynamics properties: how the system grows, self-maintains as well as the effects of perturbations, i.e. how the system reacts to antigens, collapses and reorganizes. However, integrating the heterogeneity of cell populations, phenotypes, lineages, cell location and interactions, cell differentiation across generations (16) in the different biological, and time scales, is problematic in such a mathematical form, which make these models particularly difficult to handle.

The evolution of homogeneous mathematical model of cell populations (17) toward “spatialized,” discrete, and heterogeneous software models (18) has allowed the reproduction and observation of more detailed and thus complex behaviors. For example, this made possible to model lymphocyte dynamics from thymic selection (19, 20) up to quantitative modeling of immune responses, as extensively reviewed (21) with development of agent-based and automata models (22). However, both population-based mathematical model (a top-down approach) and discrete cell-based model (a bottom-up approach) and the various platforms developed have limitations (23). Conversely, the Statecharts language (24) and the visual reactive tools (25) such as biocharts (26) and reactive animation applied to various systems (27) developed by Harel et al. are a powerful way to simulate complex dynamical biological behavior with more didactic representation than equations. Such models have revealed emergent properties during thymic differentiation (28) pancreatic islet organogenesis but also the immune response in lymph node (29).

## Lack of Interoperability, Under-Use of Software Models

Although in Immunology there is more than 20-years tradition of software and mathematical modeling, very few of them have been the object of further exploitation once published and made available (30). Models are often under-used because experimentalists can be reluctant to entertain mathematical formalization and because published models are largely disposable: rapidly forgotten after being published, instead of providing a foundation to build upon. Moreover, the various expressions of these models with different mathematical descriptions, programing languages, software libraries and graphical packages, require much effort in understanding and running the software and prevent interoperability.

## Using Visual Language to Communicate and Execute Models

Immunologists often conceptualize the dynamical evolution of their systems in terms of “state-transitions” of biological objects and do it by means of personalized and informal graphical illustrations. Thus, the adoption of a more formal and standard type of state-transition diagram could improve the current situation to not only help biologists to better understand each other but also to facilitate the production and the reading of software code executing these visual transitions, at level of populations or agents (31).

Thus, in this paper, we promote the development and usage of a visual, computational approach more comprehensible than mathematical equations and programing instructions. This should improve our understanding of lymphocyte dynamics, the exchange on this understanding and simplify the implementation of models by non-specialists delivered from the production of executable code or mathematical equations, to concentrate to *in silico* experiments.

### Level of abstraction and multi-scale modeling

A model describes a complex system from the “real word” and thus requires abstraction. This abstraction is performed as the immunologist decides about an experimental protocol in order to observe selected objects at different scales and to follow them in time and space. For example, the capacities of the immune system to preserve the homeostasis and to provide rapid adaptation to an antigen and anamnestic responses can be observed at the organism level, through physiological or pathological clinical observations that relate to lower scale levels. At molecular level, the somatic generation of the diversity of an immuno-receptor, as the TCR, allows for a dynamic network of interactions with antigens. At the cell level, this leads to clonal selection, activation and division. At the organ level, the fluidity of the system insures constant tissue redistribution of cells and molecules, cell migration from thymus to spleen and lymph nodes.

Thus, models of lymphocyte population dynamics and turnover consist in reconstructing the components or “entities” of the system across various scales, from molecules to organisms, to determine the relations/interactions through space (varying from micrometer to meters) and “processes” through time (varying between microseconds to years) as explained below. However, the formalization and abstraction of the immune objects as entities undergoing processes, with the help of spatial and dynamic ontologies, respectively defined as SNAP and SPAN (32), as well as cell/molecule interactions (33), is rarely done, maintaining a language-barrier between biologists and theoreticians. In the following, some examples will be given to help the immunologists with the transition between current mathematical models to computer ones and with the terms currently used in modeling.

### Define entities, states, location, interactions, granularity

The immune “entities” could be described according to the language used by the modeler. A cell exists in one “state”: it could be quiescent or in a given phase of the cell cycle or dead. In addition the phenotype and/or a function of a cell define a given state, as CD4 helper T cells. Cells are “located” in various tissues and are in “relation” with other entities. Finally, cells can be considered at various level of “granularity.” For example, T lymphocyte populations are “aggregation” of T cells according to criteria of phenotype, structure or function, although heterogeneity still prevails inside these populations at lower granularity. Accordingly, cells can be modeled at population level (with continuous model as ordinary equation) or at cell level according to space (with discrete model as automata or multi-agent system).

### Define processes

According to ontologies, cells participate to various processes, such as division, activation, differentiation, interaction, clonotype selection, apoptosis or migration. According to the states of the cells, their evolution can thus be modeled as “state-transition” that can be applied to various processes in parallel: for example, a thymocyte can differentiate while migrating in cortex and medulla. Note that processes at other levels like molecular or organ levels can similarly be described and modeled. Finally, all these process will determine the global cell dynamics and turnover.

## The Unified Modeling Language for High-Level Modeling

“High-level programing languages” are based on abstraction and use of natural language that is easier to understand as compared to “low level programing language,” based on codes. Thus, visual modeling language considers biological-object as conceptual abstract-objects that endure processes. The level of abstraction allowed by these diagrams makes possible to distinguish more easily the “entities” as T cells and the “processes” that occur at different levels such as differentiation, migration and cell cycle. Moreover, such “state-transition diagrams” allow computing parallel pathways at various scales to avoid redundancy that is inherent in the formal description of multi-level, heterogeneous and concurrent systems and to model heterogeneity in a very simplified and economical form (as compared to mathematical equations) (31).We have thus used the well-established Unified Modelling Language (UML – a sofware standard) that still remains approachable to the lab-immunologist, convenient for the theoretician and that can be directly adopted for the high-level graphical depiction (31, 34). The adoption of UML state-transition diagrams that transcends any programing language or computer platform, will allow both experimentalists and theorists to work together at a higher level than writing software code or mathematical equations. This final step is progressively more and more automatized out of the diagrams. Example of basic transformations of mathematical equations into state-transition diagrams including elementary, parallel, independent, or coupled state transition have been given (31), to familiarize biologists with the general approach.

## Refactoring from Low Level (Code, Equations) to High-Level (Diagram) Modeling Languages

To convince the immunologist of the feasibility of this approach as well as the benefit gained by adopting it, we sketch in the rest of the paper how existing “low level programed” immune models should gain in readability and accessibility by adopting a “high-level graphical” representation under the form of “state-transition diagrams.” We present a “refactoring” of two published models of T cell biology in the thymus. Refactoring consists in restructuring the code or equations of a model to improve its expression, readability and extensibility, without changing its external behavior. One model consists in cell population differentiation modeling with differential equations (continuous model). The other one is a discrete model. Originally it was an automata model consisting of a discrete lattice, where each site (cell) in a given state, follows some rules in space and time that depends on local neighbors (18). It has been refactorized as an agent-based model (ABM), depicting individual cell behavior through thymus differentiation and migration. It would be much too long and redundant to describe in details the behavior of these two models. We do not pretend here to modify at all the results obtained by the running of these models (the readers interested in these results are invited to access the original papers). We have just reshape them into a state-transition diagrammatic form that allows execution of simulations reproducing the original results with similar parameter values.

### Population-based model describing the conveyor-belt T cell differentiation in thymus

**Figure 1:**
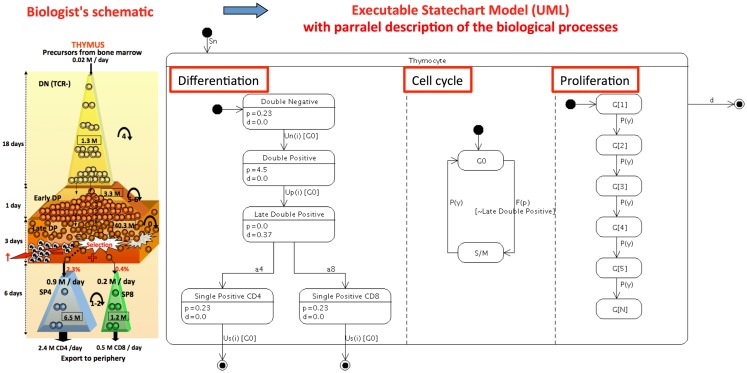
**Refactoring the continuous population (ODE) conveyor-belt model of thymocyte differentiation (8) into a computer executable visual language: The «biological schema »of thymocyte dynamics originally proposed is transformed into a UML state-transition diagram**. This diagram represents the evolution of cell populations in the thymus, represented by their state from DN to SP, with UML figuration of the input (close circle arrow) and output representing death and thymus exit (open circle arrow) and transitions (oriented arrows). Parallel processes (underlined with red box) as differentiation, cell cycle and proliferation are depicted more explicitly in this representation than with the original 30 mathematical equations. Annotation with the proliferation and death rates values indicated in each state are values from the best scenario observed in the original paper.

The original model (8) is a compartmentalized ordinary differential equation (ODE) model, rather complex to read and manage by immunologists. This model reflects the conceptual “conveyor belt” model of thymic T cell differentiation, schematically represented by immunologists by the continuous ordered transition of cells through the different stages with time (35–37). Figure 1 represents a biological schema, originally published and the “state-transition” description of the model (in a UML state-transition diagram) as it is proposed now. Although the original model is composed of 30 differential equations, the whole mathematical description and the code that captures it, can easily be deduced and regenerated from the Figure 1. Conversely, the mathematical equations can be automatically generated from the state-transition diagram as previously described (38). In essence, the model is summarized by the input, transition and output from the thymus, by “parallel processes” that occur concomitantly, as differentiation, cell cycle, proliferation and death, and by exit from the thymus. Note that these parallel processes concern various biological levels and time scales. The “differentiation” process represents each stage of the conveyor belt, from double negative (DN) to single positive (SP) cells, with flows into and from a particular stage according to the general equation:

$$dx_{i}/dt=2\gamma \;px_{i-1}-\left(p+d+u\left(i\right)\right)x_{i}$$

*p*, *d*, and *u* represent proliferation, death, and differentiation, respectively, and *x_i_* represents the *i*th stage. The model mainly consists of constant hematopoietic progenitor influx in thymus (Sn); differentiation between thymocyte developmental phenotypes DN and double-positive (DP) cells differentiation into CD4^+^ or CD8^+^ cells, then egression of SP stage, either, to the periphery [Us4(i), Us8(i)]; proliferation [Pn(y), Pp(y), and Ps(y)]; positive and negative selection (a4, a8); and natural cell death (Dn, Dp, and Ds). In parallel, the “cell cycle” is represented: the cell switches between quiescence (G0) and cycle with division (S/M). The parameter γ set to 1 represents the cell division into two daughter cells. There is the possibility to induce a perturbation into the system through the specific depletion of T cells entering the S/M cycle phase (*p*), if γ is set to 0. This represents the presence or absence of a pharmaco-genetic conditional treatment by ganciclovir that induces apoptosis related to the incorporation of a nucleotide analog during DNA elongation. This rule applies to all cell populations except in late DP quiescent cells as indicated in the schema. The “proliferation” depicts that the daughters of a proliferating cell transit into the next generational compartment, except during treatment (γ = 0) when dividing cells die by apoptosis and are lost from the model. The parameter *u* is an increasing function of generation (G1 to *n*), making cells more likely to differentiate between phenotypic compartments as they progress through the cell generations.

The parallelism in this graphical model largely simplifies the original formulation of ODEs while remaining faithful to it. Hierarchy and compound states are present again clearly reducing the diagram clutter. Other representation of the model depicting the differentiation with linear cell generations is also possible (31).

### Discrete model describing the differentiation along the migration of T cells in the thymus in a 2-D environment

**Figure 2:**
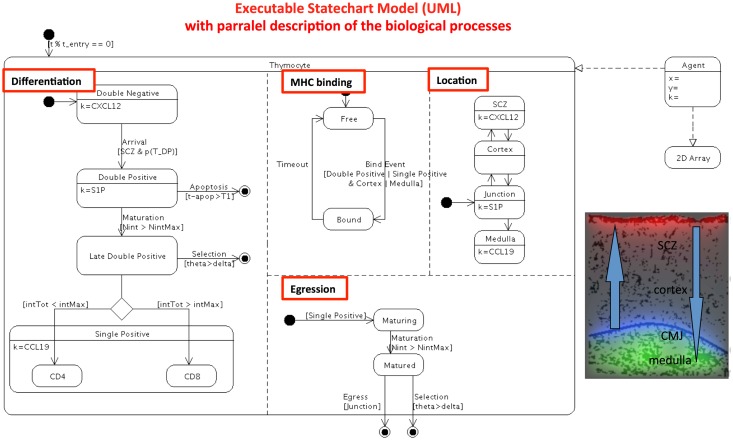
**Refactoring the automata single-cell model of thymocyte differentiation and migration (20) into a computer executable visual language**. The 40 pages of Fortran code is transformed in computer UML state-transition diagram. This diagrams describes the experimentally observable heterogeneity, and the biologically relevant parallel processes (underlined in red box). As in Figure 1, input, output, and oriented transitions are described in the state-transition diagram. Parallel state-transitions represent the evolution of single cells in the thymus, represented by their differentiation from DN to SP stage, sequential binding event of TCR/MHC peptide on epithelial cells, thymic location, egression of cell when matured. Additional qualitative abstractions for computational model of individual cells in semi-realistic environment are represented by the 2-D array: cells are allowed migrating sequentially through the epithelial cell network (black network) across the various thymic areas, guided by chemokine gradients CXCL12 (red), CCL19/CCL21 (green), S1P (blue).

The original model (20) is a discrete-based “cellular automata” computer model. The model depicts the behavior of individual thymocytes that evolve in the 2-D epithelial cell network, guided by chemokines gradients. The current model (Figure 2) is now an Agent-Based Model (ABM). Again, the interested reader is referred to the original paper for a detailed understanding of the simulation. Although available for download, the 40 pages of FORTRAN source code are far from easy to understand. After refactoring, the transition rules of any agent (thymocytes) map onto a parallel state-transition diagram. The conception of the state-transition diagram, as done here, should considerably improve the understanding of the model (even for the original programmer) allowing the researchers to progress further with the existing simulator. A complete description of the model includes additional implementation details that are abstractions of the mechanisms behind cell decisions to differentiate. The parallel state-transition diagram represents the different simultaneous transitions taking place in the model and coded as various cellular automata rules: a cell in the model as it differentiates transits in successive states, it may be bound or not to thymic epithelial cells via TCR/MHC, it moves and may be located into one of several anatomical compartments of the thymus. As indicated in the boxes, the gradient of chemokines (k) orients the migration of cells for each specific stage of differentiation, and chemokines are localized in specific areas of the thymus. A T cell sums up the time and number of interactions with the same or different epithelial cells. This sum value determines whether the T cell is positively or negatively selected. DP and SP phenotypes have their own threshold parameters. They cannot differentiate until they cross a threshold number of interactions. If, after a given time, a DP cell has not reached this threshold, it enters apoptosis by neglect. If the time is too long, it is negatively selected. A threshold parameter simulates the phenotype decision. With this “signal-duration” hypothesis, long duration TCR-MHC interactions promote the CD4 phenotype and short duration promotes the CD8 phenotype.

It is important to notice that both refactored models, population-based and ABM can now be compared, are directly executable and can provide simulations of physiology, pathologies, and treatment, while not being the scope of this paper. Moreover, their parameters can be automatically tuned to fit experimental data. Any ABM model can also be run as a population version to save time in simulation (McEwan et al., manuscript in preparation). Moreover, the flexibility of these diagrams allows assembling the parts of the biological puzzle piece after piece and improving the models.

## Perspectives

As shown here, state-transition diagrams can represent high-level semantics suitable to clarify immunological concepts and to aid communication among interdisciplinary researchers. It can also represent low levels quantitative information suitable for individual-based ABM and population-based ODE modeling. Organization of immune knowledge using a standardized, diagrammatic formal language should greatly improve knowledge integration at multi-scale levels and sharing between experimentalist and theoretician collaborators, rendering their software more readable, scalable, and usable. We are currently working on ways of automatically generating executable code out of these state-transition diagrams. State-transition diagrams supports the extension and interoperability of published models. This will help for dynamic computational modeling of lymphocyte behavior in health and diseases, and for “*in silico*” experiments to predict and explain the puzzling T cells dynamics and the effect of immunological perturbations.

## Author Contributions

Véronique Thomas-Vaslin and Hugues Bersini designed the project and wrote the article, Adrien Six and Jean-Gabriel Ganascia participated to discussions.

## Conflict of Interest Statement

The authors declare that the research was conducted in the absence of any commercial or financial relationships that could be construed as a potential conflict of interest.

## References

[1] Lavelle C Berry H Beslon G Ginelli F Giavitto J Kapoula Z From molecules to organisms: towards multiscale integrated models of biological systems. Theor Biol Insights (2008) 1:13–22

[2] Germain RN Meier-Schellersheim M Nita-Lazar A Fraser ID Systems biology in immunology: a computational modeling perspective (*). Annu Rev Immunol (2011) 29:527–85 10.1146/annurev-immunol-030409-101317 21219182 PMC3164774

[3] Thomas-Vaslin V Six A Bellier B Klatzmann D Lymphocytes dynamics repertoires, modeling. In: Dubitzky W Wolkenhauer O Cho KH Yokota H, editors. Encyclopedia of Systems Biology. New York: Springer (2013). p. 1149–62 10.1007/978-1-4419-9863-7_96

[4] Thomas-Vaslin V Six A Bellier B Klatzmann D Lymphocyte dynamics and repertoire, biological methods. In: Dubitzky W Wolkenhauer O Cho KH Yokota H, editors. Encyclopedia of Systems Biology. New York: Springer (2013). p. 1145–49 10.1007/978-1-4419-9863-7_95

[5] Asquith B Borghans JA Ganusov VV Macallan DC Lymphocyte kinetics in health and disease. Trends Immunol (2009) 30(4):182–9 10.1016/j.it.2009.07.013 19286425

[6] Ossimitz G Mrotzek M The basics of system dynamics: discrete vs. continuous modelling of time. Proceedings of the 26th International Conference of the System Dynamics Society. Wiley-Blackwell (July 2008) (2008). Available from: http://teoriasistemas.mex.tl/imagesnew/6/6/4/5/2/SimulaciondiscretavsContinua.pdf

[7] Mehr R Globerson A Perelson AS Modeling positive and negative selection and differentiation processes in the thymus. J Theor Biol (1995) 175(1):103–26 10.1006/jtbi.1995.0124 7564390

[8] Thomas-Vaslin V Altes HK de Boer RJ Klatzmann D Comprehensive assessment and mathematical modeling of T cell population dynamics and homeostasis. J Immunol (2008) 180(4):2240–50 18250431 10.4049/jimmunol.180.4.2240

[9] Bains I Thiebaut R Yates AJ Callard R Quantifying thymic export: combining models of naive T cell proliferation and TCR excision circle dynamics gives an explicit measure of thymic output. J Immunol (2009) 183(7):4329–36 10.4049/jimmunol.0900743 19734223

[10] den Braber I Mugwagwa T Vrisekoop N Westera L Mögling R de Boer AB Maintenance of peripheral naive T cells is sustained by thymus output in mice but not humans. Immunity (2012) 36(2):288–97 10.1016/j.immuni.2012.02.006 22365666

[11] Mehr R Perelson AS Blind T-cell homeostasis and the CD4/CD8 ratio in the thymus and peripheral blood. J Acquir Immune Defic Syndr Hum Retrovirol (1997) 14(5):387–98 10.1097/00042560-199704150-00001 9170412

[12] Almeida AR Amado IF Reynolds J Berges J Lythe G Molina-París C Quorum-sensing in CD4+ T cell homeostasis: a hypothesis and a model. Front Immunol (2012) 3:125 10.3389/fimmu.2012.00125 22654881 PMC3360200

[13] Chao DL Davenport MP Forrest S Perelson AS A stochastic model of cytotoxic T cell responses. J Theor Biol (2004) 228(2):227–40 10.1016/j.jtbi.2003.12.011 15094017

[14] Terry E Marvel J Arpin C Gandrillon O Crauste F Mathematical model of the primary CD8 T cell immune response: stability analysis of a nonlinear age-structured system. J Math Biol (2012) 65(2):263–91 10.1007/s00285-011-0459-8 21842166

[15] De Boer RJ Perelson AS Ribeiro RM Modelling deuterium labelling of lymphocytes with temporal and/or kinetic heterogeneity. J R Soc Interface (2012) 9(74):2191–200 10.1098/rsif.2012.0149 22513720 PMC3405764

[16] Yamanaka YJ Gierahn TM Love JC The dynamic lives of T cells: new approaches and themes. Trends Immunol (2013) 34(2):59–66 10.1016/j.it.2012.10.006 23200626 PMC3565007

[17] Louzoun Y The evolution of mathematical immunology. Immunol Rev (2007) 216:9–20 17367331 10.1111/j.1600-065X.2006.00495.x

[18] Celada F Seiden PE A computer model of cellular interactions in the immune system. Immunol Today (1992) 13:56 10.1016/0167-5699(92)90135-T 1575893

[19] Morpurgo D Serentha R Seiden PE Celada F Modelling thymic functions in a cellular automaton. Int Immunol (1995) 7(4):505–16 10.1093/intimm/7.4.505 7547676

[20] Souza-e-Silva H Savino W Feijoo RA Vasconcelos AT A cellular automata-based mathematical model for thymocyte development. PLoS One (2009) 4(12):e8233 10.1371/journal.pone.0008233 20011042 PMC2784945

[21] Cohn M Mata J Quantitative modeling of immune responses. Immunol Rev (2007) 216(1):5–8 17367330 10.1111/j.1600-065X.2006.00492.x

[22] Chavali AK Gianchandani EP Tung KS Lawrence MB Peirce SM Papin JA Characterizing emergent properties of immunological systems with multi-cellular rule-based computational modeling. Trends Immunol (2008) 29(12):589–99 10.1016/j.it.2008.08.006 18964301

[23] Bianca C Pennisi M Immune system modelling by top-down and bottom-up approaches. Int Math Forum (2012) 7(3):109–28

[24] Harel D Statecharts: a visual formalism for complex systems. Sci Comput Program (1987) 8(3):231–74 10.1016/0167-6423(87)90035-9

[25] Efroni S Harel D Cohen IR Toward rigorous comprehension of biological complexity: modeling, execution, and visualization of thymic T-cell maturation. Genome Res (2003) 13(11):2485–97 10.1101/gr.1215303 14597657 PMC403768

[26] Kugler H Larjo A Harel D Biocharts: a visual formalism for complex biological systems. J R Soc Interface (2010) 7(48):1015–24 10.1098/rsif.2009.0457 20022895 PMC2880074

[27] Vainas O Harel D Cohen IR Efroni S Reactive animation: from piecemeal experimentation to reactive biological systems. Autoimmunity (2011) 44(4):271–81 10.3109/08916934.2010.523260 21244340

[28] Efroni S Harel D Cohen IR Emergent dynamics of thymocyte development and lineage determination. PLoS Comput Biol (2007) 3(1):e13 10.1371/journal.pcbi.0030013 17257050 PMC1782042

[29] Swerdlin NI Cohen IR Harel D The lymph node B cell immune response: dynamic analysis in-silico. Proc IEEE (2008) 96(8):1421–43 10.1109/JPROC.2008.925435

[30] Bersini H, editor. Object-oriented refactoring of existing immune models. In: *Artificial Immune Systems. Lecture notes in Computer Science* Berlin: Springer-Verlag (2009). p. 27–40.

[31] Bersini H Klatzmann D Six A Thomas-Vaslin V State-transition diagrams for biologists. PLoS One (2012) 7(7):e41165 10.1371/journal.pone.0041165 22844438 PMC3402529

[32] Grenon P Smith B SNAP and SPAN: towards dynamic spatial ontology. Spat Cogn Comput (2004) 4(1):69–104 10.1207/s15427633scc0401_5

[33] Pappalardo F Lefranc MP Lollini PL Motta S A novel paradigm for cell and molecule interaction ontology: from the CMM model to IMGT-ONTOLOGY. Immunome Res (2010) 6(1):1 10.1186/1745-7580-6-1 20167082 PMC2834662

[34] Bersini H UML for ABM. J Artif Soc Soc Simul (2012) 15(1):

[35] Penit C Vasseur F Sequential events in thymocyte differentiation and thymus regeneration revealed by a combination of bromodeoxyuridine DNA labeling and antimitotic drug treatment. J Immunol (1988) 140(10):3315–23 3258880

[36] Penit C Lucas B Vasseur F Cell expansion and growth arrest phases during the transition from precursor (CD4-8-) to immature (CD4+8+) thymocytes in normal and genetically modified mice. J Immunol (1995) 154(10):5103–13 7730616

[37] Scollay R Godfrey D Thymic emigration: conveyor belts or lucky dips? Immunol Today (1995) 16:268–74 10.1016/0167-5699(95)80179-0 7662096

[38] McEwan CH Bersini H Klatzmann D Thomas-Vaslin V Six A. A computational technique to scale mathematical models towards complex heterogeneous systems. COSMOS Workshop ECAL 2011 Conference; Paris: Luniver Press (2011).

